# Techno-economic analysis of a novel laccase production process utilizing perennial biomass and the aqueous phase of bio-oil

**DOI:** 10.1186/s40643-025-00924-2

**Published:** 2025-07-31

**Authors:** Elmin Rahic, Nicholas Cassady, Kurt Rosentrater

**Affiliations:** 1 Bioeconomy Institute, Iowa State University, 617 Bissell Rd, Ames, IA 50011 USA; 2 Department of Biochemistry, Biophysics and Molecular Biology, Iowa State University, Ames, IA 50011 USA; 3 Department of Agricultural and Biosystems Engineering, Iowa State University, Ames, IA 50011 USA

**Keywords:** Laccase, Techno-economic analysis, Optimization, Solid-state fermentation, Bio-oil, Aqueous phase, Prairie, Perennial, Biomass

## Abstract

This study explored a novel laccase production process utilizing perennial biomass and the aqueous phase from bio-oil. Experimental and techno-economic modeling were coupled to gain insight into and optimize the solid-state fermentation conditions for minimizing the laccase selling price. At relatively small scales, processing only 230 Mg of prairie biomass per year, a 5-year return on investment required a minimum laccase selling price of $0.05/kU, assuming a 10% discount rate. The associated sensitivity analysis showed the minimum laccase selling price to be most sensitive to parameters affecting laccase output from the plant, which included the number of batches produced per year and laccase recovery assumptions. Limitations and future outlooks were provided, emphasizing the need for further process optimization to minimize fermentation time and downstream losses.

**Supplementary Information:**

The online version contains supplementary material available at 10.1186/s40643-025-00924-2.

## Graphical Abstract

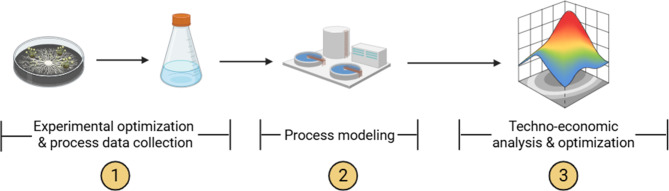

## Introduction

Fast pyrolysis depolymerizes biomass into three fractions: gas, char, and bio-oil. Carbon dioxide, carbon monoxide, and methane are the primary components contained in the gaseous fraction, which is often used for process heating (Zhang et al. 2023). The char consists mostly of inert carbon and ash, which has many potential applications, including bioremediation, anaerobic digestion, and as a soil amendment (El-Naggar et al. 2019; Patel et al. 2022; Zhou et al. 2020). Lastly, the bio-oil is a tar-like material made of various anhydrosugars, aromatic compounds, and more, and can be upgraded into a renewable petroleum alternative (Lachos-Perez et al. 2023).

To facilitate the upgrading of bio-oil into renewable products, the aqueous fraction (hereafter referred to as the aqueous phase, AP) can be separated from the valuable heavy ends through stage fractionation processes (Pollard et al. 2012). AP is predominantly composed of water and carboxylic acids, but may also contain various alcohols and low molecular weight lignin fractions, like phenols (Liang et al. 2013). These characteristics make AP unsuitable for many common upgrading techniques that are better suited for handling the higher molecular weight “heavy ends” components. However, AP has been identified as a potential feedstock for microbial bioprocessing due to its high organic content. Specifically, previous studies have attempted to utilize AP as a carbon source for heterotrophic fermentation, through anaerobic digestion, microalgae cultivation, or other means (Zhao et al. 2016; Zhou et al. 2019). While these studies offer promising insights towards the valorization of AP, its inherent toxicity has proven difficult to overcome, despite the use of detoxification measures.

Recently, AP was reported to be an effective inducer for laccase production in the white-rot fungus, *Pleurotus ostreatus* (Rahic 2024). Laccase is a multi-copper oxidase found naturally in plants, fungi, and bacteria. In plants, laccase plays a role in lignin polymerization, whereas in fungi, laccase often assists in lignin depolymerization reactions (Dwivedi et al. 2011). Further, bacterial laccases are generally more active and stable at higher temperatures and pH than fungal laccases, but are often produced in smaller quantities and have a lower redox potential (Janusz et al. 2020). Nevertheless, laccase is capable of catalyzing oxidation reactions on a wide range of substrates. As a result, laccase has been studied for its potential application in a myriad of industries, including food and beverage production, textiles, bioremediation, and bioenergy (Mayolo-Deloisa et al. 2020).

Laccase can be produced from submerged or solid-state fermentation. Solid-state fermentation may offer several advantages for white-rot fungi, as it better mimics the environmental conditions in which the fungi naturally grow. Additionally, solid-state fermentation can require less process water, making it potentially more sustainable than submerged culturing methods. While many studies have investigated laccase production from white-rot fungi using solid-state fermentation; reports on its economic viability have been limited.

Previously, laccase production was studied using prairie biomass as the substrate for solid-state fermentation (Rahic 2024). When integrated into agricultural landscapes, prairie offers numerous ecological services, including improved nutrient, soil, and water retention, while also providing wildlife habitat (Schulte et al. 2017). However, a financial incentive may be necessary to encourage farmers to implement these perennials into their farming operation. As a lignocellulosic biomass, prairie has the potential to be utilized in biorefining processes, yet very few studies have evaluated valorization strategies for prairie (Olafasakin et al. 2024; Rahic et al. 2024; Wild et al. 2025). Thus, this study aims to conduct a techno-economic analysis (TEA) investigating the viability of a novel laccase production method, simultaneously valorizing both AP and perennial biomass.

## Materials and methods

### Substrate and inoculum origin

The prairie biomass used in this study originated from the Iowa State University Comparison of Biofuel Cropping Systems research plots, seeded with a mixture of ~ 59 different species. Kordbacheh et al. (2019) provides details on prairie speciation. The *P. ostreatus* grain inoculum used in this study was purchased from Field & Forest Products. The inoculum was stored at 4 °C upon delivery and used within one week. The AP used in this study was derived from the fast pyrolysis of corn stover in a fluidized bed reactor performed according to Polin et al. (2019). The AP was subjected to overliming treatment prior to use, following the method detailed by Zhao et al. (2015). Relevant characteristics of prairie biomass and treated AP are provided in Table 1.

**Table 1:** Characteristics of the prairie biomass and treated AP used for this study

Characteristic	Unit	Prairie biomass	AP
Water	% wt	6.1	43.8
Volatile Solids	% wt	86	-
Lignin	% wt	16.7	-
Hemicellulose	% wt	24.9	-
Cellulose	% wt	31.3	-
pH		-	9.93
Total phenolics	g/L gallic acid equiv.	-	15.3

### Growth nutrient screening for *Pleurotus ostreatus*

Nutrient screening was performed by cultivating *P. ostreatus* on plates containing: 20 g/L agar, 10 g/L finely milled prairie biomass, 4 g/L KH_2_PO_4_, 1 g/L MgSO_4_, 0.2 g/L CaCl_2_, 0.2 g/L CuSO_4_, and a nitrogen-containing nutrient at a concentration of 5 g/L, unless otherwise specified. Four nutrients were tested: corn steep solids, peptone, yeast-extract, and ammonium sulfate. Each plate was inoculated with a 1 cm mycelial plug of *P. ostreatus* grown on potato-dextrose agar at 28 °C for 7 days. The nutrient screening plates were incubated at 28 °C for four days, followed by harvesting of the mycelial biomass by scraping and quantifying the growth gravimetrically. Each condition was evaluated in triplicate.

### Two-stage fermentation for laccase production

Laccase production from *P. ostreatus* was conducted following the protocol by Rahic (2024) with modifications. In short, the fermentation process was divided into two stages: growth stage and induction stage. During the growth stage, *P. ostreatus* was cultivated in rectangular LDPE containers to mimic a tray bioreactor. Each container held sterilized prairie biomass moistened to 80% moisture content with liquid media containing: 10 g/L corn steep solids, 4 g/L KH_2_PO_4_, 1 g/L MgSO_4_, 0.2 g/L CaCL_2_, and 0.2 g/L CuSO_4_. Commercial grain spawn of *P. ostreatus* was used as inoculum. The quantity of substrate and inoculum used, as well as the growth stage time, was based on the experimental design, described in greater detail in Sect. “Economic analysis”. After the growth stage, the fungal culture was transferred to 500 mL Erlenmeyer flasks and submerged with non-sterile water to 94% moisture content. AP and CuSO_4_ were added at 2.5% (v/v) and 1.1 g/L, respectively, and the cultures were held on orbital shakers at 25 °C with 155 rpm agitation.

### Analyses

Laccase activity was measured calorimetrically through the oxidation of 0.2 mM 2,2’-azino-bis(3-ethylbenzothiazoline-6-sulfonic acid) (ABTS) (ε = 36,000 M^− 1^ cm^− 1^) at 420 nm in 0.1 M citrate-phosphate buffer (pH 3) at 25 °C. Calorimetric determination of laccase activity is the most commonly used method reported in literature (Baltierra-Trejo et al. 2015; and was calculated following the suggested equation by Baltierra-Trejo et al. (2015) and reported in units per gram of prairie biomass. One unit of laccase activity was defined as the quantity of laccase required to oxidize 1 µmol of ABTS per min. Total phenolic content was measured using the folin-ciocalteu method. Prairie biomass samples were submitted to Celignis Analytical (Limerick, Ireland) for lignin, cellulose, and hemicellulose for analysis.

### Techno-economic analysis

**Fig. 1:**
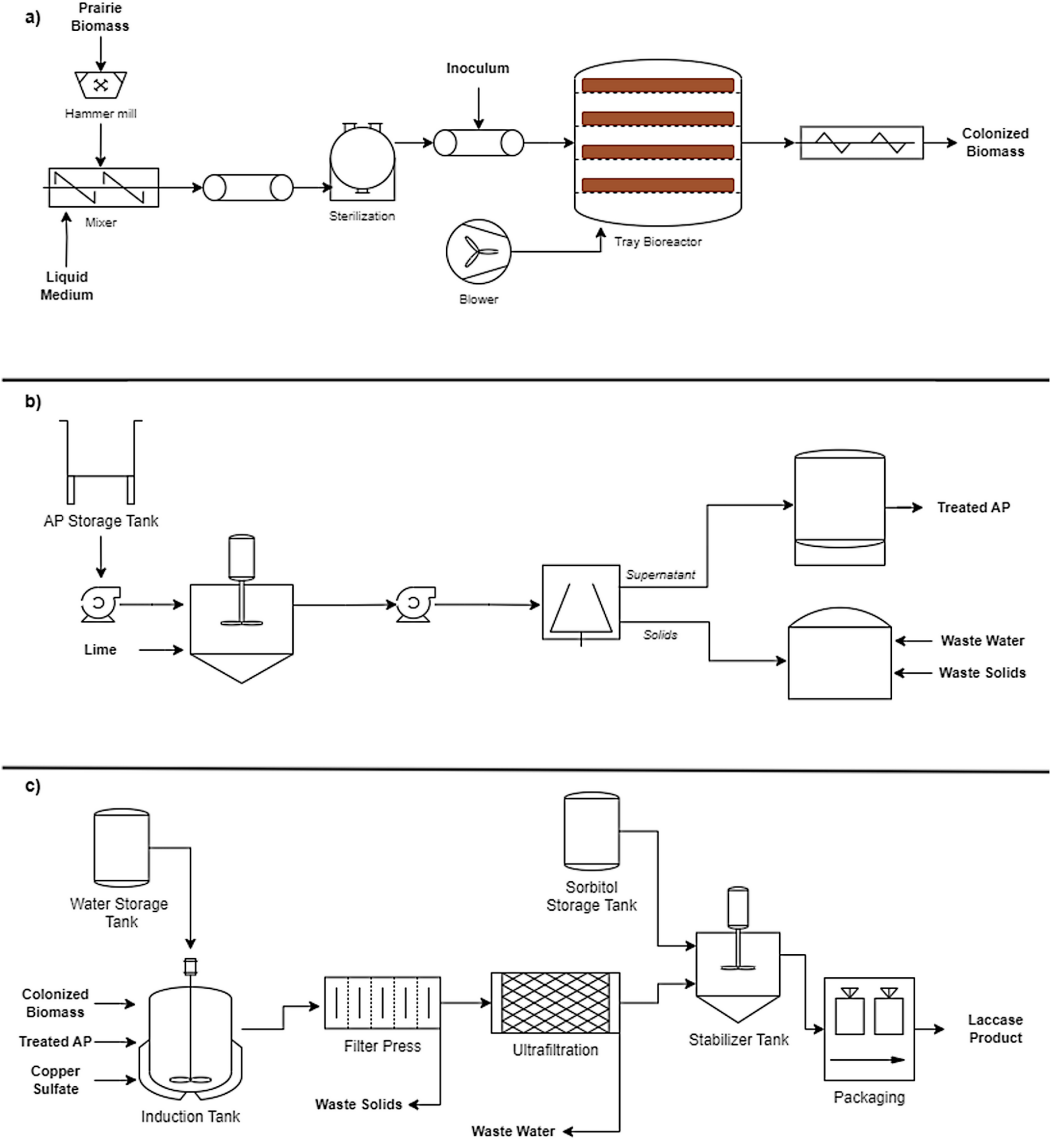
Process flow diagram showing **a**) upstream and fungal growth-stage processes, **b**) AP preparation, and **c**) downstream processing

Spreadsheet modeling of the laccase production facility was performed for TEA (Supplementary Information). Experimental data from this study, as well as existing literature, were used to inform mass and energy flows for the model. Laccase production was modeled as a batch process, depicted in Fig. 1.

The process is divided into three stages: growth stage, induction stage, and downstream processing. The growth stage begins with mixing and preparation of the prairie biomass with liquid media to achieve a final moisture content of 80% (Fig. 1a). The substrate mixture is then sterilized prior to inoculation. Once inoculated, the fungus is cultured statically at 28 °C in a tray bioreactor. Meanwhile, AP is treated using the overliming method outlined in Sect. “Substrate and inoculum origin” (Fig. 1b). Once the growth stage is complete, the colonized biomass is loaded into a mixing tank and submerged with water, CuSO_4_, and AP, following the exact parameters outlined in Sect. “Two-stage fermentation for laccase production” (Fig. 1c). The mixture is mixed continuously, during which laccase is produced extracellularly. After the induction phase, the mixture is subjected to filtration by a plate and frame filter press, where it is assumed that 10% of the laccase is lost to the solids fraction. After filtration, the laccase solution is concentrated via ultrafiltration. Zaccaria et al. (2019) reported a volumetric concentration factor of 60 with 183% laccase activity recovery. Meanwhile, Bryjak & Rekuć (2010) reported a concentration factor of ~ 27 with a ~ 90% laccase activity recovery. Thus, an average concentration factor of 43.5 and the more conservative laccase activity recovery of 90% were used for the TEA. Afterward, the laccase solution is mixed with sorbitol (33% v/v) as a stabilizing agent and assumed to be packaged in one-gallon jugs. An additional 25% loss in laccase activity is assumed from storage and shipping, for a final laccase recovery of 60.6%. No water reuse or valorization of side streams was considered for this analysis.

The laccase production plant was scaled based on the quantity of prairie substrate used, from 2 to 15 Mg/batch. Plant construction time was set to 24 months with a 6-month start-up time at 50% capacity. The plant was assumed to operate for 7,446 h per year, equivalent to an 85% capacity factor, for a plant lifetime of 20 years. Given the long fermentation time, the plant was assumed to run on a concurrent schedule around the rate-limiting operation. Specifically, preparations for a new batch are assumed to begin during the longest process of the current batch. This maximizes the number of batches that can be completed per year. However, this study assumes only 5 concurrent batches can be completed before the scheduling must restart. The full spreadsheet model with all associated assumptions can be found in the Supplementary Information.

### Economic analysis

#### Optimization of laccase production parameters

Given the lack of information regarding commercial laccase prices, this laccase production facility was evaluated based on its minimum laccase selling price (MLSP). MLSP was calculated as the price needed to achieve a 5-year break-even period (after construction) assuming a 10% discount rate.

Laccase yield and induction stage time for the model were evaluated experimentally based on a central composite design studying three factors: substrate bed depth, substrate:inoculum (S:I) ratio, and growth stage time (Table 2). The laccase yield and induction time were not modeled as responses; rather, the experimental results were implemented directly into the process model to model the MLSP at each scale. Table 2 lists the experimental conditions for each run. The three factors were correlated through a second-order polynomial equation:

1 $$\eqalign{ Y{\rm{ }} & = {\rm{ }}{\beta _0} + {\rm{ }}{\beta _i}{x_i} + {\rm{ }}{\beta _j}{x_j} + {\rm{ }}{\beta _k}{x_k}{\beta _{ii}}{x_i}^2 \\&+ {\rm{ }}{\beta _{jj}}{x_j}^2 + {\rm{ }}{\beta _{kk}}{x_k}^2 + {\rm{ }}{\beta _{ij}}{x_i}{x_j} \\& + {\rm{ }}{\beta _{ik}}{x_i}{x_k} + {\rm{ }}{\beta _{jk}}{x_i}{x_k} \cr}$$

Where Y is the predicted MLSP, β are coefficients estimated by the model, and x_i_, x_j_, and x_k_ represent the variables: substrate bed depth, S:I ratio, and growth stage time, respectively. The model was generated using non-linear regression and the significance was evaluated using the F-test. All statistical analyses were performed using JMP16.

**Table 2:** Central composite design with minimum laccase selling price (MLSP) as the response

Run	Factors			MLSP ($/kU) ^a^				
	Bed depth (cm)	S:I ratio (g/g)	Time (days)	2 Mg/batch	4 Mg/batch	8 Mg/batch	10 Mg/batch	15 Mg/batch
1	2	2.75	6	0.119	0.095	0.078	0.075	0.070
2	2.41	2.01	4.81	0.142	0.113	0.091	0.087	0.081
3	2.41	2.01	7.19	0.122	0.097	0.078	0.075	0.070
4	2.41	3.49	4.81	0.154	0.123	0.099	0.095	0.088
5	2.41	3.49	7.19	0.121	0.096	0.078	0.074	0.069
6	3	2	6	0.135	0.105	0.085	0.080	0.075
7	3	3	4	0.214	0.166	0.134	0.127	0.117
8	3	3	6	0.151	0.117	0.095	0.089	0.083
9	3	3	6	0.147	0.114	0.092	0.087	0.081
10	3	3	6	0.158	0.123	0.099	0.094	0.087
11	3	3	6	0.159	0.124	0.100	0.095	0.087
12	3	3	8	0.140	0.109	0.088	0.083	0.077
13	3	4	6	0.134	0.105	0.084	0.080	0.074
14	3.59	2.01	4.81	0.140	0.107	0.086	0.082	0.076
15	3.59	2.01	7.19	0.121	0.093	0.075	0.071	0.066
16	3.59	3.49	4.81	0.147	0.112	0.090	0.086	0.079
17	3.59	3.49	7.19	0.128	0.097	0.078	0.075	0.069
18	4	2.75	6	0.112	0.087	0.070	0.067	0.062

a. Price represented as USD per kilo unit (kU) (1,000 laccase units)

#### Capital costs

The total capital investment is defined as the sum of all equipment purchase costs, multiplied by the Lang Factor. As a solid-liquid processing plant, a Lang Factor of 4.9 was chosen for this process model (Verret et al. 2020). Temporal adjustments for purchase costs were made based on the Chemical Engineering Plant Cost Index (CEPCI), assuming a current CEPCI of 798. Equation 2 shows the formula used to calculate purchase costs.

2 $$\eqalign{ Purchase\;cost & = Reference\;cost \\& \times {\left({{{Designed\;capacity} \over {Reference\;capacity}}} \right)^n} \\& \times \;\left({{{Current\;CEPCI} \over {Reference\;CEPCI}}} \right) \cr}$$

Here, $\:n$ represents the scaling factor to include economies of scale. For equipment with unknown scaling factors, a factor of 0.6 was assumed (Williams 1947).

Information on solid-state bioreactors is scarce, particularly for tray bioreactors. As such, the bioreactor purchase price was based off a packed-bed bioreactor (Vasco-Correa and Shah 2019) with the following size and cost adjustments. The trays are assumed to be 15’ x 45’ with two feet of vertical space between each tray, which includes empty space and the height of the substrate on each tray. The maximum volume (including empty space) for one bioreactor is 500 m^3^, with a 70% working volume. To account for automation or additional complexities, an additional two-times multiplier was applied to the bioreactor cost. The blower size was calculated based on the airflow rate used by Manan and Webb (2020) and adjusted based on bioreactor size. Detailed breakdown on individual equipment costs can be found in the Supplementary Information.

#### Operating costs

Table 3 lists the assumptions and breakdown of the annual operating costs for the laccase production plant. The operating costs include materials, utilities, labor, and facilities. The inoculum cost was estimated from Field & Forest (2024), which was the source of inoculum used in the experimental portion of this study. The prairie biomass and AP were assumed to cost $150/Mg and $500/m^3^, respectively. Although the cost of the prairie biomass may be high relative to other lignocellulosic biomass (Billion-Ton Report, 2023), prairie is assumed to be grown in replacement of commodity crops, such as corn or wheat, suggesting that the prairie biomass should be valued at a higher cost relative to conventional feedstocks. Given the uncertainty in price for the biomass and AP, their cost was included for sensitivity analysis. Chemical prices were based on various commercial vendor prices. Further information on materials cost can be found in the Supplementary Information. Labor cost is dependent on plant size, with a minimum of four full-time operators assumed, plus one operator for every additional tray bioreactor unit needed. Wages are set at $30/hr, with an additional 45% to account for benefits.

**Table 3:** Component assumptions for operational costs

Parameters	Cost	Source
**Materials Cost (C**_m_ **)**		
Prairie biomass	$150/Mg	Assumed
Corn steep	$0.50/kg	Alibaba (2024)
K_2_HPO_4_	$1/kg	Molbase (2024)
CuSO_4_ * 5H2O	$8.4/kg	Molbase (2024)
CaCl_2_	$0.17/kg	Molbase (2024)
MgSO_4_ * 7H2O	$0.09/kg	Alibaba (2024)
Water	$0.03/Mg	Assumed
Inoculum	*Calculated*	Field and Forest (2024)
Raw AP	$500/m3	Assumed
CaOH (lime)	$120/Mg	Alibaba (2024)
70% Sorbitol	$760/Mg	ChemAnalyst (2024)
Packaging	$0.58/bottle	Alibaba (2024)
**Utilities Cost (C**_**u**_ **)**		
Electricity	$0.07/kWh	Rosentrater and Zhang (2021)
Steam	$12/Mg	Poliafico (2007)
**Labor Cost (C**_**l**_ **)**		
Wage	$30/hr	Assumed
Benefits factor	0.45	Assumed
**Facility Costs (C**_**f**_ **)**		
Maintenance	0.02xCPC	Peters et al. (2003)
Depreciation	Straight-line method	Internal Revenue Service
Property tax & insurance	0.01xTCI	Assumed

#### Discounted cash flow analysis

The discounted cash flow analysis was performed by considering the cash flow based on the time value of money. The construction period was assumed to last two years, followed by a six-month start-up period. As such, a negative cash flow is assumed for the first two years. The plant was modeled to run for 20 years total. The discount rate was set at 10% with a 40% income tax (Yang and Rosentrater 2019). All equipment and material costs are presented in 2024 U.S. dollars.

#### Sensitivity analysis

Sensitivity analysis is a useful tool to evaluate the effect of individual parameters on the profitability of a plant. Seven parameters were chosen for sensitivity analysis: prairie biomass cost, AP cost, inoculum cost, number of batches completed per year, operating cost, capital cost, and laccase activity loss. These parameters were chosen based on their level of uncertainty and assumed impact. Table 4 lists the individual parameters for each scenario, as well as their optimistic and pessimistic ranges.

**Table 4:** Parameters to be evaluated for sensitivity analysis

Parameter	Pessimistic	Base	Optimistic
Prairie biomass cost ($/Mg)	250	150	75
Inoculum cost	+ 50%	-	-50%
Laccase activity loss (% loss)	60	40	30
# Batches per year	+ 25%	-	-25%
AP cost ($/m^3^)	750	500	250
Operating cost	+ 25%	-	-25%
Capital Cost	+ 25%	-	-25%

## Results

### Preliminary evaluation of nutrients to enhance *Pleurotus ostreatus* growth

**Fig. 2:**
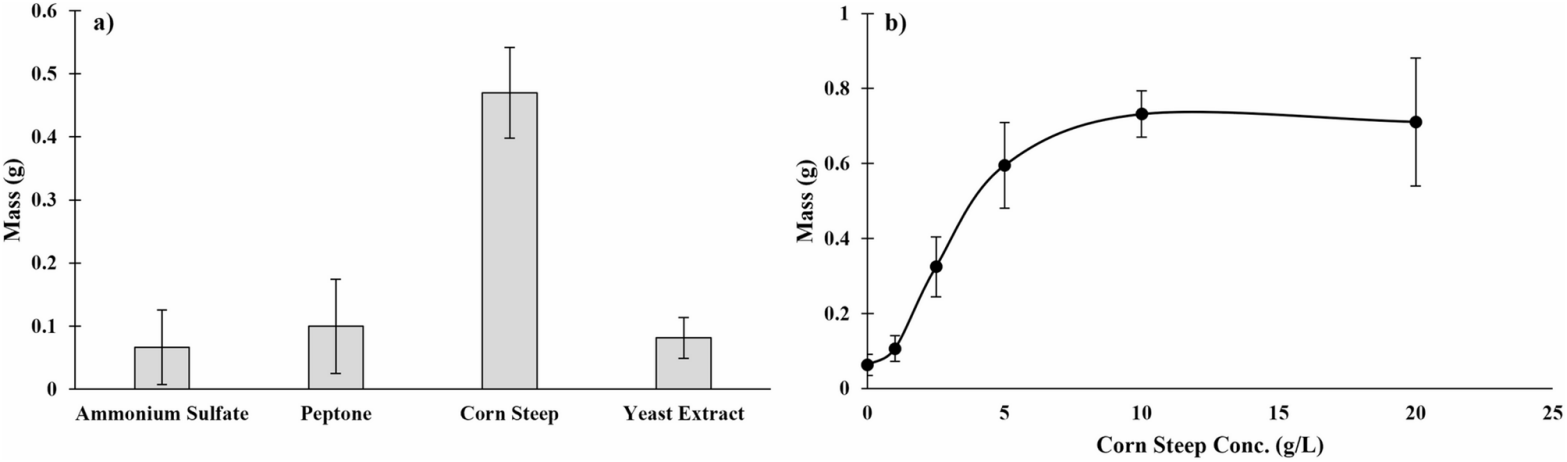
Evaluation of growth rate of *P. ostreatus* on agar media by **a**) comparison of different nitrogen sources, and **b**) evaluating different concentrations of corn steep. Data is presented as means of three replicates, with error bars representing standard deviations

Prior to the economic assessment of laccase production from *P. ostreatus*, four nitrogen-containing nutrients were screened to evaluate their effect on mycelial growth in an effort to improve the fungus’s growth rate on prairie biomass. As shown in Fig. 2a, corn steep resulted in the greatest growth for *P. ostreatus.* Meanwhile, growth differences between ammonium sulfate, peptone, and yeast extract were insignificant (*p* > 0.05). Figure 2b depicts the mycelial growth of *P. ostreatus* at different corn steep concentrations. Corn steep addition improved the growth of *P. ostreatus* up to a concentration of 10 g/L but showed no improvement in growth at higher concentrations. As such, corn steep solids were included in the media at 10 g/L in the following work.

### Economic optimization of growth stage parameters

In addition to nutrient medium composition, it is important to study the effects of other design and operational parameters for solid-state fermentation. The effect of substrate bed depth, S:I ratio, and growth time were evaluated experimentally with regards to laccase production yields and total fermentation time using a central composite design (Table 1). Table 5 provides the experimental laccase yields and induction stage time for each condition evaluated. The results indicate the substrate bed depth to be the most significant factor affecting laccase yield (*p* < 0.05), with lower bed depths often resulting in greater laccase yields, particularly when the S:I ratio is high. This is expected, as greater bed depths may result in substrate compaction, limiting the mass transfer of gases (i.e. oxygen). This is also supported by studying the resulting induction stage time needed to achieve these laccase yields. At greater bed depths, longer growth times were needed to achieve optimal laccase yields and shorter induction stage times, while with shorter bed depths, the effect of growth time was less significant. These experimental results (Table 5) were incorporated into the techno-economic model to model MLSP.

**Table 5:** Experimental results for laccase yield and induction stage time used for process modeling

ID#	Factors			Results	
	Bed depth (cm)	S: I ratio (g/g)	Growth time (days)	Laccase yield (U/g)	Induction stage time (hrs)
1	2	2.75	6	964.4	257.5
2	2.41	2.01	4.81	842.5	284.5
3	2.41	2.01	7.19	844.1	227.0
4	2.41	3.49	4.81	765.6	284.5
5	2.41	3.49	7.19	1202.2	349.0
6	3	2	6	811.8	257.5
7	3	3	4	759.9	424.5
8	3	3	6	713.8	257.5
9	3	3	6	583.3	195.0
10	3	3	6	678.4	257.5
11	3	3	6	674.5	257.5
12	3	3	8	603.9	113.5
13	3	4	6	793.8	257.5
14	3.59	2.01	4.81	796.7	284.5
15	3.59	2.01	7.19	793.8	227.0
16	3.59	3.49	4.81	751.6	284.5
17	3.59	3.49	7.19	643.9	188.5
18	4	2.75	6	891.4	258.0

Table 6 shows the parameter estimates for Eq. (1) and corresponding significance levels for the base scenario. Growth time, as well as all quadratic terms showed statistical significance, while none of the interactive variables were deemed insignificant within this boundary.

**Table 6:** Parameter estimates and statistical significance values for Eq. (1) at 8 Mg/batch

Coefficient	Variable	Estimate	*F*-value	*p*-value
β0	Intercept	-0.108	> 100	< 0.0001
βi	Bed Depth	0.139	1.90	0.206
βj	S:I Ratio	0.103	0.362	0.564
βk	Time	-0.04	36.9	0.0003
βii	Bed Depth^2^	-0.026	28.9	0.0007
βjj	S:I Ratio^2^	-0.015	10.1	0.013
βkk	Time^2^	0.003	5.74	0.0435
βij	Bed Depth · S:I Ratio	-0.0004	0.004	0.954
βik	Bed Depth · Time	0.0019	0.42	0.534
βjk	S:I Ratio · Time	-0.0016	0.28	0.610

Table 7 shows the predicted optimal conditions to minimize the MLSP at each scale. At each scale, the model R^2^ was 0.92 with an insignificant lack-of-fit test (*p* > 0.05), suggesting adequate model reliability. Maximizing the substrate bed height was optimal for each scale evaluated, as it reduced cost by minimizing reactor volume; however, the optimal S:I ratio and growth time were scale dependent. At 2, 10, and 15 Mg/batch scales, the optimized conditions were nearly identical, showing a lower S:I ratio to be advantageous. At 4 and 8 Mg/batch, a higher S:I ratio was slightly more advantageous. Meanwhile, the optimal growth stage time only differed substantially for the 8 Mg/batch plant.

**Table 7:** Optimal growth stage parameters for minimizing the minimum laccase selling price (MLSP)

	Optimized Conditions			MLSP	
Scale (Mg/batch)	Bed Height (cm)	S:I Ratio (g/g)	Growth Time (days)	Predicted ($/kU) ^a^	Validated ^b^ ($/kU)
2	4	1.5	6.83	0.0793	0.0847
4	4	4	6.77	0.0599	0.0657
8	4	4	7.44	0.0483	0.0508
10	4	1.5	6.86	0.0475	0.0509
15	4	1.5	6.86	0.0439	0.047

a. Price is provided in USD per kilo unit (kU) (1,000 laccase units)

b. MLSP after performing validation experiments under the predicted optimal conditions

**Fig. 3:**
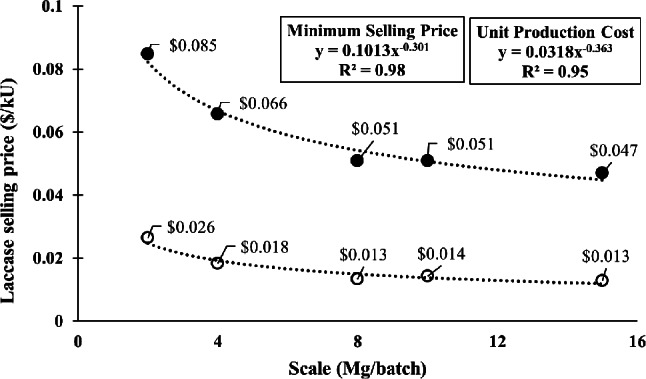
Unit production cost and minimum laccase selling price (MLSP) at different scales. Price is provided in USD per kilo unit (kU) (1,000 laccase units). Scales are represented as the quantity of prairie biomass used for fermentation per batch. Empty circles represent the unit production cost, while the filled circles represent the minimum selling price

The fermentation was repeated under the predicted optimal conditions for validation. The validated results were within 5.2–9.7% of the predicted results, with the 8 Mg/batch scale having the closest fit. Figure 3 depicts the relationship between scale and both MLSP and unit production cost for the validated results. As scale increases, MLSP and unit production costs generally decrease, but scaling beyond 8 Mg/batch provided only marginal reductions in cost and price. As such, 8 Mg/batch was chosen as the base scenario for this study.

### Capital and operating costs

**Fig. 4:**
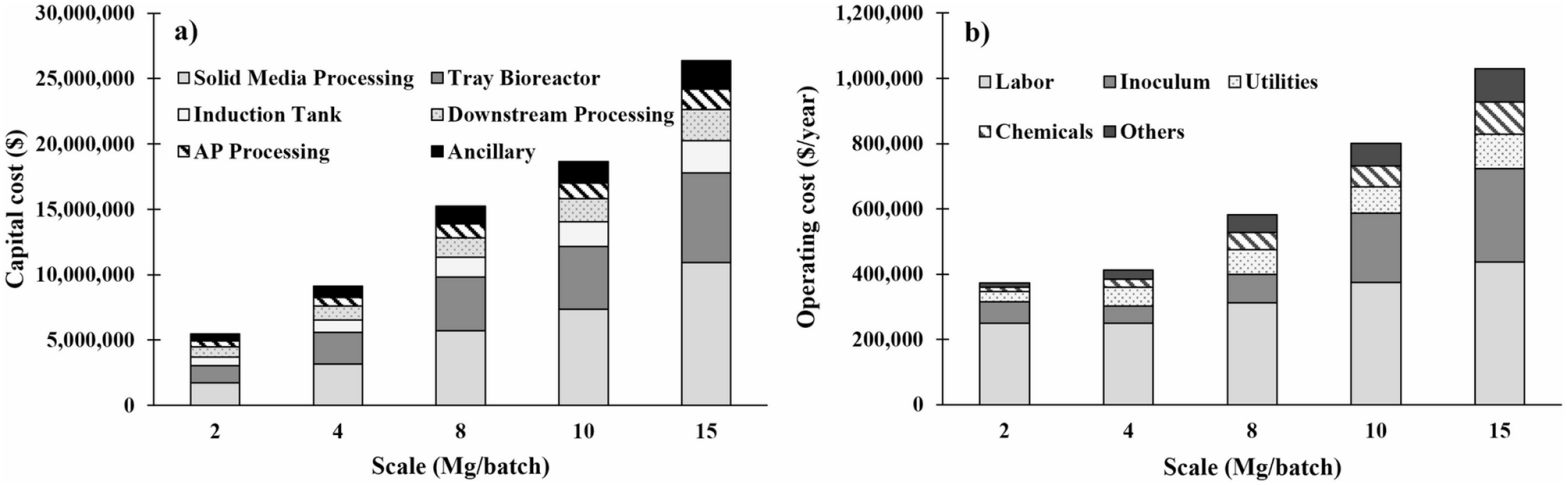
Comparison and breakdown of **a**) total capital investment and **b**) annual operating variable costs at different scales. Scales are represented as quantity of prairie biomass used for fermentation

Figure 4 provides a breakdown of the total capital and annual operating costs for the optimized conditions outlined in Table 6. Under the base scale, the total capital and annual variable operating costs are estimated at 15.2 and 1.06 million USD. Solid media processing equipment and the tray bioreactor are the largest contributors to capital cost at each scale. The costs for solid media processing are largely attributed to the sterilization unit. Labor was the largest operating cost, comprising 42–67% of the variable operating costs, but did not increase proportionally based on scale. At scales of 2–8 Mg/batch, all non-labor operating costs were relatively comparable with each other. Beyond that, the inoculum cost began to greatly increase relative to the other costs, largely due to the reduction in S:I ratio assumed for those scales (Table 6).

### Discounted cash flow analysis

**Fig. 5:**
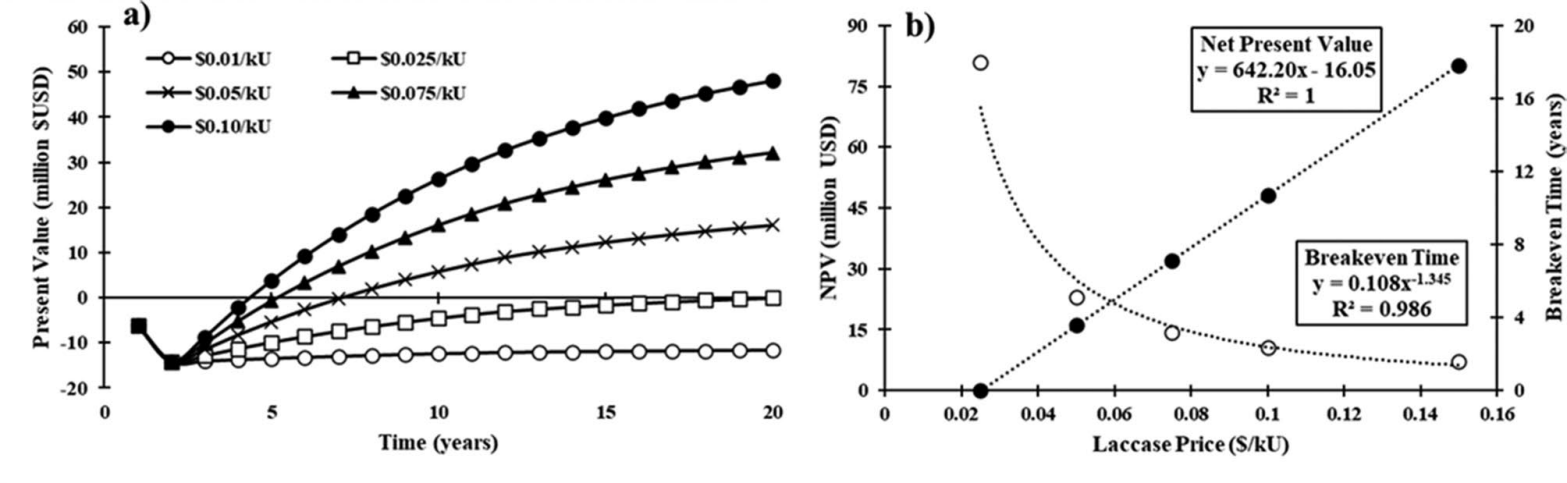
**a**) Discounted cash flow analysis under different laccase price assumptions under the base scale (8 Mg/batch). **b**) Relationship between laccase selling price and the 20-year net present value (NPV) and breakeven time of the facility at the base scale. Hollow circles represent breakeven time, while filled circles represent the NPV. Breakeven time does not account for construction. All prices are provided in USD per kilo unit (kU) (1,000 laccase units)

Figure 5 illustrates the cumulative discounted cash flow (after tax) at the base scale over the plant life. Given that there are no publicly available commercial selling prices for laccase enzymes, the selling price was adjusted to explore the potential cash flow returns under different pricing scenarios. As expected, greater laccase prices resulted in greater cash flows and shorter breakeven times for the facility. The relationship between the selling price and 20-year net present value (NPV) was linear (Fig. 5b), but its relationship with the breakeven time was not. A minimum of $0.025/kU was needed to achieve a net positive return within the plant life.

### Sensitivity analysis

**Fig. 6:**
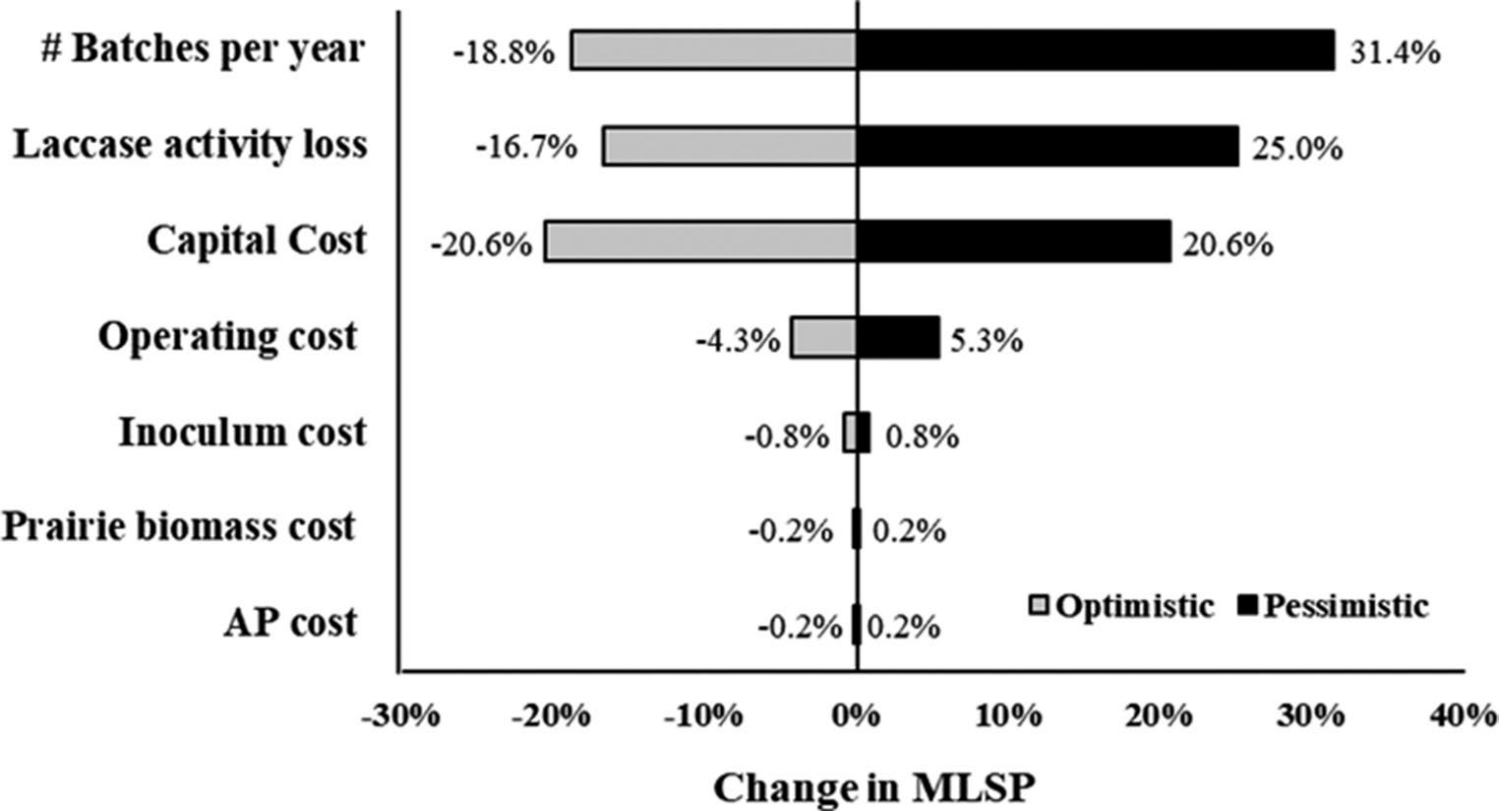
Sensitivity analysis of different parameters at a scale of 8 Mg/batch based on pessimistic and optimistic assumptions defined in Table 4. MLSP represents the minimum laccase selling price

Figure 6 depicts the sensitivity of MLSP to changes in individual parameters under the base scenario of 8 Mg/batch. The prairie biomass, inoculum, and AP costs were insignificant, resulting in a < 1% change in MLSP. This is likely due to the relatively low quantities of these resources used at this scale. Changes to the operating cost had a slight effect on MLSP, while changes to capital costs had more significant effects on MLSP. The MLSP also showed high sensitivity to changes in laccase output from the production facility, as changes to laccase activity losses and the number of batches produced annually were among the most significant factors.

## Discussion, limitations, and future outlook

Given the novelty of this laccase production method, it is likely that many process improvements can be made to reduce costs, increase product output, or decrease product losses. However, this study provides critical insights into the current, preliminary prospects of this process system. Table 8 presents a summary of the process conditions and economic outlook for the base scenario at 8 Mg of biomass per batch.

**Table 8:** Process simulation summary for an 8 Mg/batch plant

Parameter	Scenario
Total capital investment ($)	15,241,822
Annual operating cost ($)	1,059,097
Batches (#/year)	28.2
Laccase yield (kU/year) ^a^	149.9 × 10^6^
MLSP ($/kU)	0.051
Discounted ROI	109%
IRR (after tax)	24.5%
NPV ($)	16,574,382

a. Yield represented in kilo units (kU) (1,000 laccase units)

At this scale, approximately 28 batches are generated annually, with the largest scheduling bottlenecks being the growth stage and induction stage of fermentation. While the concurrent scheduling method allowed more batches to be completed annually, the total number of batches completed per year was still limited. To achieve a five-year return-on-investment (ROI) at this scale, an MLSP of $0.05/kU was needed, assuming a 10% discount rate.

The two-stage fermentation process produced impressive laccase yields when compared to literature sources on submerged or solid-state fermentation using *P. ostreatus*. For example, in this study, a laccase yield of 1097 U/g (~ 86,400 U/L) was obtained under optimized conditions. Most papers report laccase yields ranging from 12,200 to 74,000 U/L (Bakratsas et al. 2024; Diaz et al. 2010; Mazumder et al. 2009; Téllez-Téllez et al. 2008; Tinoco et al. 2011; Tlecuitl-Beristain et al. 2008; Zhao 2017). However, as Baltierra-Trejo et al. (2015) reported, there is a lack of standardization in how laccase activity is calculated in literature, making it difficult to compare yields across different studies.

It is difficult to assess whether the MLSP would be competitive in the market given the lack of publicly available data on commercial laccase prices, comparisons can be made with laboratory-grade, “off-the-shelf” prices, with the understanding that these may cost more per unit of enzyme relative to bulk prices. Brugnari et al. (2021) reported a range of literature estimates and off-the-shelf laccase prices between 0.40 and 155 $/kU, which is between 8 and 3,100 times greater than the MLSP estimated in this study. This indicates that, given the early-stage nature of this process, this novel production method shows promise for cost-competitive laccase production.

Despite the relatively sparce commercial implementations of laccase, Zerva et al. (2019) have demonstrated growing market interest in these enzymes from both the academic and industrial sectors for textiles, food and beverage, biofuels, and paper. Our previous study determined that this specific laccase may be effective in the decolorization of certain dyes and pretreating biomass for greater cellulose digestibility (Rahic 2024). Specifically, the study investigated laccase additions of 4–40 U/L of dye solution, and 8–80 U/g of biomass for pretreatment. Using these reported results, and assuming a laccase cost of $0.05/kU, a treatment cost of $0.20 – $2.00/m^3^ of dye solution ($10 – $100/mol of dye), and $400 – $4,000/Mg of biomass is obtained. While this laccase may not offer a viable option for biomass pretreatment, the resulting cost for dye removal falls within the typical range of other methods evaluated in literature (Ighalo et al. 2022). Future research directions could further investigate market applications for this novel laccase and explore laccases produced from other species using similar production methods.

The fermentation process was modeled based on experimental data gathered from a three-factor central composite design (Table 2), showing a significant effect from the growth stage fermentation parameters on the MLSP. The optimal configurations for the fermentation parameters were found to be scale dependent. For all scales evaluated, maximizing the substrate bed height (4 cm) was optimal for decreasing MLSP. Bed height is a particularly important consideration for tray bioreactors, as larger bed sizes allow for higher throughput and smaller bioreactors and facility sizes, thus reducing costs. However, increasing the bed height generates more compaction of the biomass, which can prevent airflow from passing through the substrate (Pitol et al. 2016), thereby limiting microbial growth. The optimal S:I ratio was determined to be at the outer boundary of the experimental design, either 1.5–4 g/g, depending on scale. It should be noted that the difference in MLSP at the low and high boundaries were minor. Lastly, the optimal growth stage time ranged from 6.77 to 7.44 days depending on scale. This is because the longer growth times typically resulted in increased laccase yields (Supplementary Information) without becoming the rate limiting process for the laccase production plant. Further investigation of the fermentation process should focus on the following: (1) evaluating the relationship between substrate particle size and bed height to minimize bioreactor size without limiting fungal growth, (2) increasing the S:I ratio to minimize the cost of inoculum needed for fermentation, and (3) reducing the total fermentation time to increase the number of batches produced annually. Additional research in these areas could provide substantial economic benefits.

The laccase production system is estimated to be a capital-intensive process (Fig. 4a), similar to other solid-state bioprocesses reported (Hafid et al. 2021; Lin et al. 2019; Vasco-Correa and Shah 2019). The capital cost breakdown was consistent for each scale, with solid media processing and the tray bioreactor system incurring the largest costs. Together, the cost for the tray bioreactor system and autoclave contribute to 43–52% of the total capital investment, demonstrating the need to innovate and reduce size or costs for this technology. The operating cost breakdown differed slightly depending on scale. For all plant sizes evaluated, labor contributed the most to operating costs. At 10 and 15 Mg/batch scales, the inoculum cost increased significantly, mostly due to the low S:I ratio used at those scales requiring a greater quantity of inoculum. In this study, the inoculum was assumed to be purchased through a commercial vendor; however, producing the inoculum on-site could lower this cost at the expense of higher capital investment. Although energy use was not explicitly optimized in this study, the process benefits from lower energy demands during fermentation due to low temperature (28 °C) demands during fermentation. The total electricity use estimated in this study, per unit of enzyme production, was 5.3 Wh/kU.

Several limitations for this process model should be taken into consideration. First, this study employs lab-scale data that may differ from production-scale operations. Additionally, data availability for solid-state bioprocessing equipment is scarce, and thus, difficult to model. However, TEA is often rife with uncertainty and variability, particularly for technologies at low technological readiness levels (van der Spek et al. 2017). One advantage of TEA for early-stage technologies is that design parameters can be evaluated under a consistent set of assumptions, which was the objective for the experimental work in this study. Although some optimization work was performed, it is of the authors’ opinion that significant improvements to this laccase production system are still possible.

Region-specific limitations may also exist for this fermentation process. While prairie biomass could be substituted for other, more abundant, lignocellulosic biomass, this may result in differences in laccase yield and fermentation conditions. As stated previously, prairie biomass was chosen for the various environmental and ecological benefits that perennials can provide. However, prairie biomass may not be available in all regions, so alternative feedstocks should be evaluated for this process as well.

With additional research, significant improvements in plant design and economics could be achieved. First, reductions in fermentation time would greatly increase the number of batches produced annually (Fig. 6). Currently, the rate-limiting operation is the induction stage of fermentation. Optimizing the environmental conditions for the induction stage may substantially lower the duration for laccase production. Based on the sensitivity analysis (Fig. 6), laccase recovery significantly impacts MLSP, suggesting that further research and optimization of downstream processes should be done while considering the different applications for laccase enzymes. Integration of the process model with other processes may also enhance the economics and help build more circular systems. For example, after solid-liquid separation, the solid substrate can be utilized as a feedstock for composting or anaerobic digestion (Gao et al. 2021; Lou et al. 2017; Vasilakis et al. 2023), which may provide added revenue to the plant. Additionally, process water could be reused to decrease the cost of waste storage and disposal.

## Conclusions

This study evaluated the economic viability of an early-stage, novel laccase production process that provides a valorization strategy for perennial biomass and bio-oil aqueous phase. A process model was generated based on experimental data to model and optimize the effect of three fermentation parameters on the economics of a laccase production plant. At a base scale of 8 Mg perennial biomass per batch, 28 batches can be generated each year, achieving a 24.5% internal rate of return at a laccase selling price of $0.05/kU. Sensitivity analysis showed laccase recovery and the annual number of batches to be the most significant towards impacting the MLSP. Future efforts should be dedicated towards fermentation optimization and generating pilot scale data on downstream processing and solid-state fermentation technologies.

## Supplementary Information

Below is the link to the electronic supplementary material.

Supplementary Material 1

## Data Availability

Data is available in Supplementary Information. Any additional data will be made available upon request.

## Abbreviations

AP: Bio-oil aqueous phase
TEA: Techno-economic analysis
ABTS: 2,2’-azino-bis(3-ethylbenzothiazoline-6-sulfonic acid)
CEPCI: Chemical Engineering Plant Cost Index
S: I: Substrate: inoculum ratio
MLSP: Minimum laccase selling price
NPV: Net present value
ROI: Return on investment
